# Phenol‐Catalyzed Discharge in the Aprotic Lithium‐Oxygen Battery

**DOI:** 10.1002/anie.201702432

**Published:** 2017-05-10

**Authors:** Xiangwen Gao, Zarko P. Jovanov, Yuhui Chen, Lee R. Johnson, Peter G. Bruce

**Affiliations:** ^1^ Departments of Materials and ChemistryUniversity of OxfordParks RoadOxfordOX1 3PHUK

**Keywords:** electrochemistry, lithium-air battery, lithium-O_2_ battery, phase-transfer catalyst, phenol

## Abstract

Discharge in the lithium‐O_2_ battery is known to occur either by a solution mechanism, which enables high capacity and rates, or a surface mechanism, which passivates the electrode surface and limits performance. The development of strategies to promote solution‐phase discharge in stable electrolyte solutions is a central challenge for development of the lithium‐O_2_ battery. Here we show that the introduction of the protic additive phenol to ethers can promote a solution‐phase discharge mechanism. Phenol acts as a phase‐transfer catalyst, dissolving the product Li_2_O_2_, avoiding electrode passivation and forming large particles of Li_2_O_2_ product—vital requirements for high performance. As a result, we demonstrate capacities of over 9 mAh cm^−2^_areal_, which is a 35‐fold increase in capacity compared to without phenol. We show that the critical requirement is the strength of the conjugate base such that an equilibrium exists between protonation of the base and protonation of Li_2_O_2_.

The rechargeable Li‐O_2_ battery has been the focus of intense research in recent years due to its high theoretical specific energy, and its position as one of but a few technologies able to exceed the performance of popular lithium‐ion systems.^1^ The Li‐O_2_ battery is noted for its potential application in electrified vehicles, however, major challenges must first be overcome and these span problems surrounding rate, capacity, stability of cell components,^2^ and the development of metallic negative electrodes. Addressing each of these will take substantial effort from a range of disciplines. The aprotic Li‐O_2_ battery is composed of a lithium metal anode separated by an aprotic electrolyte solution from the cathode, which consists of a porous O_2_ electrode. On discharge, O_2_ is reduced to Li_2_O_2_ at the cathode, the reverse oxidation taking place on charge, and it is this reaction that defines the battery and its associated challenges.^3^ Other reactions have been recently proposed, but here we focus on formation of Li_2_O_2_.^4^

Li_2_O_2_ can form either directly at the electrode surface or from a chemical step in solution following the first reduction, where the two routes are termed the surface mechanism and solution mechanism, respectively.3a Li_2_O_2_ is an insoluble, insulating solid and therefore discharge by the surface mechanism results in low capacities, poor rates and early cell death.3a,3f, 5 Whereas, if Li_2_O_2_ forms via the solution mechanism high capacities can be obtained and early cell death by surface passivation is avoided. In aprotic electrolytes, it is the solubility of the intermediate LiO_2_ that controls the discharge route, and this in turn is dependent on the solvent properties, where high donor number and acceptor number solvents strongly solvate the Li^+^ and O_2_^−^ ion, respectively.3a, 6 Unfortunately, solvents containing polar groups able to promote a solution mechanism are also unstable towards reactive intermediates.^7^ There is consequently a need to develop methods of promoting a solution mechanism during discharge while using stable, low polarity solvents.

A number of strategies aimed at achieving a solution mechanism in stable solvents have been developed. High DN salts have been shown to increase the capacity 4‐fold.6a, 8 Redox shuttles are able to transfer electrons from the electrode to O_2_ in solution thus forming LiO_2_ away from the electrode surface.^9^ Phthalocyanines and DBBQ are able to catalyze O_2_ reduction directly to Li_2_O_2_, and in the latter case without contact with the electrode resulting in significant improvements in capacity.^10^ In all cases when using additives to promote a solution mechanism, it will likely be necessary to protect metallic lithium with a solid‐state separator to avoid crossover from the region of the cathode to the anode, where the mediators would be reduced.^11^

Recently it has been shown that fortuitous ingress of water into the battery is responsible for enhanced capacities in many reports, suggesting that protic additives can also promote a solution mechanism.3d,3f, 12 Here we demonstrate that addition of the weak acid phenol to an ether‐based electrolyte (tetraethylene glycol dimethyl ether, TEGDME) within the Li‐O_2_ battery is able to promote discharge via a solution mechanism. We propose that this occurs by action of the proton, which dissolves Li_2_O_2_ as proposed by Gasteiger et al.,3d and in a similar way to that reported in Na‐O_2_.12d The proton of phenol behaves as a phase‐transfer catalyst, able to chemically convert the insoluble discharge product Li_2_O_2_ to its soluble protonated analogue, which subsequently redeposits again from solution to form large Li_2_O_2_ particles—a requirement for high capacity, high rate batteries. Using phenol as an additive, we are able to demonstrate capacities of over 9 mAh cm^−2^_areal_, which is a 35‐fold increase in capacity compared to without phenol. Our work demonstrates the importance of the conjugate base when using protic additives.

In order to explore the effect of phenol on oxygen reduction in the Li‐O_2_ battery, cyclic voltammetry was carried out. Figure 1 a shows voltammetry for O_2_ reduction in a TBATFSI‐based electrolyte (lithium‐free) showing the well known quasi‐reversible CV,^13^ with addition of phenol resulting in a shift to positive potentials and an increase in peak height. Phenol has been used in aprotic solvents as a proton source during fundamental studies of the oxygen reduction reaction.^13^, ^14^ The CV shown in Figure 1 a is similar to that seen in previous reports but here we observe a positive shift on addition of phenol. This is consistent with a proton coupled electron transfer (PCET) forming OOH, rather than formation of O_2_^−^ reported in other solvents. We note that previous studies were performed in higher DN solvents that are likely to stabilize H^+^ more than TEGDME. The H^+^ will be associated with strongest base, in this case the phenolate ion. The enhanced peak height is due to an overall 2 e^−^ product, by a second reduction or a disproportionation to H_2_O_2_ as previously reported.^13^, ^14^ The reverse peak is now due to oxidation of H_2_O_2_, rather than O_2_^−^, and occurs at 3.2 V. Equations (1), (2), (3) show the complete reaction steps for the lithium‐free system.

(1) $$O{}_{2(\mathrm{sol})}+e{}^{-}+\mathrm{AH}{}_{\left(\mathrm{sol}\right)}\to \mathrm{HOO}{}_{\left(\mathrm{sol}\right)}+A{}^{-}$$

(2) $$\mathrm{HOO}{}_{\left(\mathrm{sol}\right)}+e{}^{-}+\mathrm{AH}{}_{\left(\mathrm{sol}\right)}\to H{}_{2}O{}_{2(\mathrm{sol})}+A{}^{-}$$

(3) $$2\;\mathrm{HOO}{}_{\left(\mathrm{sol}\right)}\to H{}_{2}O{}_{2(\mathrm{sol})}+O{}_{2(\mathrm{sol})}$$

**Figure 1:**
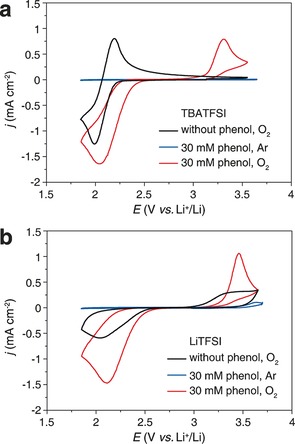
CVs for O_2_ reduction without phenol (black) and with 30 mm phenol (red) in a) 0.5 m TBATFSI in TEGDME and b) 0.5 m LiTFSI in TEGDME under O_2_. GC electrode. Scan rate 100 mV s^−1^.

Oxygen reduction in Li^+^ containing TEGDME (phenol‐free) results in the CV shown in Figure 1 b. The reaction mechanism for this process is well known and given by Equations (4), (5), (6), 3e, 15

(4) $$O{}_{2(\mathrm{sol})}+\mathrm{Li}{}^{+}{}_{\left(\mathrm{sol}\right)}+e{}^{-}\to \mathrm{LiO}{}_{2}{}^{*}$$

(5) $$\mathrm{LiO}{}_{2}{}^{*}+\mathrm{Li}{}^{+}{}_{\left(\mathrm{sol}\right)}+e{}^{-}\to \mathrm{Li}{}_{2}O{}_{2}{}^{*}$$

(6) $$2\;\mathrm{LiO}{}_{2}{}^{*}\to \mathrm{Li}{}_{2}O{}_{2}{}^{*}+O{}_{2(\mathrm{sol})}$$

and written in the form of the surface mechanism which is expected to be dominant in glycol ethers with a LiTFSI salt.3a, 6a The peak height is lower than in the equivalent CV containing TBATFSI even though a 2 e^−^ product is now forming and this is due to passivation of the electrode surface by Li_2_O_2_—a consequence of the surface mechanism.6a Addition of phenol to this system results in an increase in current on reduction, Figure 1 b, which indicates that the previously insoluble insulating Li oxides formed on reduction are able to escape into solution due to protonation to LiOOH and H_2_O_2_. In practice the result is a complex mix of reactions (1)–(3), (4)–(6) and related equilibriums.

Cells containing TEGDME saturated with O_2_ (under 1 atm of O_2_) were each discharged at several different areal current densities with and without phenol (Figure 2). In the absence of phenol, the cells died rapidly, exhibiting very small capacities and poor rate capability, in accord with previous observations and the expected performance for a cell discharging by a surface mechanism (0.1 mAh cm^−2^_areal_, equivalent to 0.09 mg of Li_2_O_2_).1c, 6a, 16 The cells with phenol discharged under the same conditions exhibited a dramatic improvement, delivering up to 35‐fold higher discharge capacities before end of life (9.1 mAh cm^−2^_areal_, equivalent to 7.8 mg of Li_2_O_2_). This demonstrates the ability of the phenol‐catalyzed discharge to overcome the limits of electrode surface area. In a practical battery this will enable low carbon fractions in the cathode—a prerequisite for high performance.

**Figure 2:**
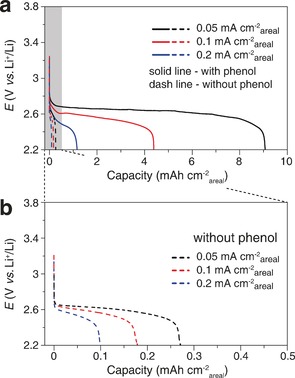
Load curves of O_2_ reduction at a gas diffusion electrode discharged in 1 m LiTFSI in TEGDME with 30 mm phenol (solid lines) and without phenol (dashed lines) under O_2_ at various areal current densities from 0.05 mA cm^−2^ to 0.2 mA cm^−2^. b) Enlarged section of the load curves recorded without phenol in (a).

The cathodes discharged with and without phenol were extracted and examined by SEM. In the absence of phenol, a film of Li_2_O_2_ was observed (Figure 3 b) consistent with a surface mechanism.3d,3f In contrast, the cells containing phenol contained larger particles of discharge product in the pores of the carbon electrode (Figure 3 d). At half discharge hemispherical particles of product were observed and no surface film was evident (Figure 3 c). At the end of discharge, the entire carbon structure close to the O_2_ reservoir was coated in similar spherical particles. Such a structure is inconsistent with a surface mechanism, which would be limited to a 7 nm thick layer due to the poor conductivity of Li_2_O_2_.^17^ The results confirm a solution mechanism is induced by phenol.

**Figure 3:**
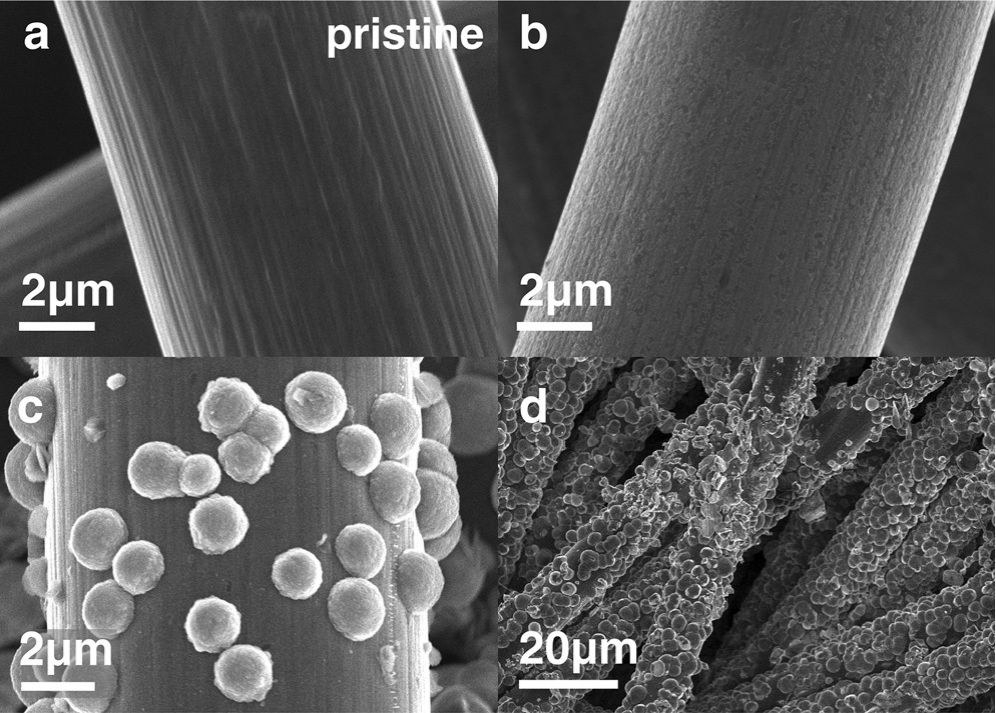
SEM images showing the Li_2_O_2_ morphologies on discharge in 30 mm phenol/1 m LiTFSI in TEGDME under O_2_. a) Pristine GDL, b) without phenol, c) half and d) full discharge with 30 mm phenol.

It is well established that electrolyte and component stability is a major challenge in Li‐O_2_ cells.^2^ A particular concern when introducing a protic additive is the promotion of acid catalyzed or coupled side‐reactions. To demonstrate the particles observed in SEM are indeed Li_2_O_2_, powder X‐ray diffraction (PXRD) and infrared spectrometry (IR) were carried out on the discharge cathode (Figure 4). Analysis shows that the major product was Li_2_O_2_, as indicated by the characteristic peaks in the FTIR spectrum and the XRD pattern. Furthermore, in situ differential electrochemical mass spectrometry (DEMS) was carried out to investigate the gas consumption on discharge (Figure 5). No other gases were detected such as CO_2_. The ratio of electrons to oxygen consumed was 2.04 e^−^/O_2_. A value close to 2 e^−^/O_2_ indicates the dominant formation of a two electron reduction product of oxygen, typically Li_2_O_2_ as indicated in the FTIR and XRD analysis.2b, 18 The yield of Li_2_O_2_, calculated by chemical analysis of the Li_2_O_2_ discharge product against TiOSO_4_, was 62 %.3d This compares to a value of 58 % without the acid, which is comparable to previous yields using GDL electrodes and indicates that phenol does not induce further side reactions.3d However, it should be noted that regardless of the presence of phenol, ethers are unstable in the battery and identification of a new solvent is essential. We note that the yield mainly depends on the carbon used and discharge with and without phenol using Vulcan C X72R carbon resulted in a yield of ca. 90 %. Here we use a GDL electrode to highlight the effect of a solution mediated discharge. Taking together, the PXRD, IR, DEMS and yield measurements indicate that the dominant discharge product in the presence of phenol in TEGDME is Li_2_O_2_ and that it forms large particles in the pores rather than thin films on the electrode surface.

**Figure 4:**
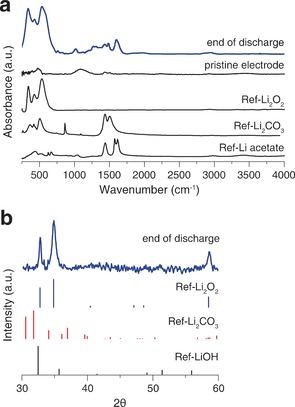
Characterization of the discharge product confirming that Li_2_O_2_ is dominant. a) Infrared spectrum and b) PXRD pattern of a GDL discharged in 30 mm phenol/1 m LiTFSI in TEGDME under O_2_.

**Figure 5:**
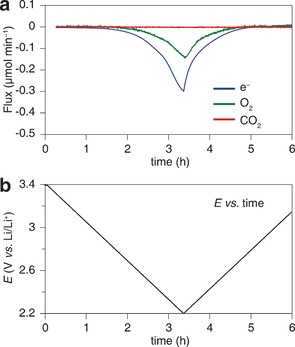
In situ DEMS of a GDL on discharge in phenol–LiTFSI–TEGDME showing 2.04 e^−^ per O_2_ consumed, consistent with Li_2_O_2_ formation. a) Discharge current (blue), O_2_ consumption (green) and CO_2_ evolution (red) in 30 mm phenol/1 m LiTFSI in TEGDME. b) Voltage profile of the DEMS cell. Cyclic voltammetry was applied at a scan rate of 0.1 mV s^−1^.

All data show that the phenol is acting as a phase‐transfer catalyst able to greatly enhance a solution mechanism during discharge. Catalysis, where the H^+^ is regenerated is confirmed by the discharge capacity of 9.1 mAh cm^−2^_areal_, which is far in excess of that possible based on phenol alone by Equations (1)–(3) due to its low concentration, 0.96 mAh cm^−2^. In contrast, discharge in the presence of perchloric acid (Figure S1 in the Supporting Information) does not result in a similar increase in capacity. Instead, a new plateau at positive potentials is observed, followed by the expected plateau for Li_2_O_2_ formation. This effect has been shown previously by Schwenke et al. who suggest that this is due to the dominant formation of H_2_O_2_ only during the first plateau,3d by reactions shown in Equations (1)–(3), until all acid is consumed, after which aprotic Li‐O_2_ chemistry takes over and a second plateau is observed, Equations (4)–(6). The result is little or no benefit in capacity or apparent catalysis. These data indicate that when acting as a phase transfer catalyst, the nature of the protic additive plays a critical role.

When discharge occurs in the presence of a weak acid, O_2_ reduction proceeds largely by formation of Li_2_O_2_. This is due to the greater Li^+^ activity compared to the H^+^ activity. If this were not the case, one would expect a positive shift in the current onset potential in Figure 1 b upon addition of phenol. An equilibrium then exists between solid and dissolved peroxide.

(7) $$\mathrm{Li}{}_{2}O{}_{2(s)}+\mathrm{AH}{}_{\left(\mathrm{sol}\right)}⇌\mathrm{HOOLi}{}_{\left(\mathrm{sol}\right)}+\mathrm{Li}{}^{+}{}_{\left(\mathrm{sol}\right)}+A{}^{-}{}_{\left(\mathrm{sol}\right)}$$

A similar reaction will exist for the superoxide case. We can confirm this step by showing that addition of phenol to solid Li_2_O_2_ in TEGDME results in an increase in soluble peroxide (Figure S2). Again, invoking the Li^+^ activity, this equilibrium is shifted to the left favoring Li_2_O_2_. The formation of HOOLi, even in small amounts, allows Li_2_O_2_ to dissolve into solution and recrystallize as larger particles by an Ostwald ripening process. This is tantamount to a solution mechanism and possesses all of the associated benefits: clean electrode surface, higher rates, larger capacity.3a

Scheme 1 compares the discharge mechanism with a weak and strong acid. The critical requirement for the acid to act as a PTC is the ability of the conjugate base, A^−^, to readily accept a proton from HOOLi such that Equation (7) lies to the left. If this is not the case then protonation will effectively be irreversible and H_2_O_2_ will form, i.e., as observed when using perchloric acid (Scheme 1). With a relatively strong base such as the phenolate ion, the proton can reversibly transfer between LiOO^−^ and phenolate, becoming catalytic and greatly enhancing the performance of the battery. In practice, the strongest base in the system will control proton activity and the equilibrium position. The concentration of Li^+^ and H^+^ and the nature of the electrolyte (salt and solvent) will all play a role.

**Scheme 1:**
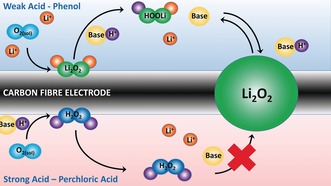
Schematic comparing the action of a strong and weak acid during discharge in a lithium‐O_2_ cell. With a strong acid (weak conjugate base), the major product is H_2_O_2_ and exchange of the H^+^ and Li^+^ is inhibited by the poor base. With a weak acid (strong conjugate base) the major product is Li_2_O_2_ and exchange of H^+^ between Li_2_O_2_ and the conjugate base is facile enabling PTC and a solution mechanism.

In summary, our work has shown that protic phase‐transfer catalysts for Li‐O_2_ batteries must be selected such that a balance is struck between protonation of the discharge product to induce solubility, and removal of the proton by the conjugate base and recrystallization of Li_2_O_2_ particles. The critical component in this equilibrium is the strength of the conjugate base, which must be able to remove a proton from LiOOH, regenerating the catalyst. Here we show that in the glycol ether, TEGDME, phenol meets these requirements and is able to induce a solution mechanism by acting as a phase‐transfer catalyst. The result is a greatly enhanced capacity (35‐fold increase) at higher rates and growth of large deposits of Li_2_O_2_ far in excess of that possible in the absence of phenol. However, it remains to be seen if such additives could be used over a longer term or if they introduce parasitic side‐reactions. Unfortunately, no stable solvent has been identified, highlighting the need for development in this area. Our work demonstrates that protic additives in combination with new solvents could be a promising method of enhancing the performance in the lithium‐O_2_ battery.

## Conflict of interest

The authors declare no conflict of interest.

## Supporting information

As a service to our authors and readers, this journal provides supporting information supplied by the authors. Such materials are peer reviewed and may be re‐organized for online delivery, but are not copy‐edited or typeset. Technical support issues arising from supporting information (other than missing files) should be addressed to the authors.

Supplementary
