# Supplementary material for: Phenol‐Catalyzed Discharge in the Aprotic Lithium‐Oxygen Battery
Source: Angew Chem Int Ed Engl. 2017 May 10;56(23):6539–43. doi: 10.1002/anie.201702432 (PMC5488210; doi:10.1002/anie.201702432)
Supplement: Supplementary file 1 — Supplementary [file ANIE-56-6539-s001.pdf]

Angewandte Chemie
Eine Zeitschrift der Gesellschaft Deutscher Chemiker

Supporting Information

Phenol-Catalyzed Discharge in the Aprotic Lithium-Oxygen Battery

*Xiangwen Gao, Zarko P. Jovanov, Yuhui Chen, Lee R. Johnson, and Peter G. Bruce**

anie_201702432_sm_miscellaneous_information.pdf

Supplementary Information

Materials and methods

Tetraethylene glycol dimethyl ether (TEGDME, Aldrich) was distilled over benzophenone and sodium under vacuum. For 500 ml of ether solvent, 9 g of benzophenone and 1.5 g of sodium were used. Distilled solvents were further dried for several days over freshly activated molecular sieves (type 4Å, Aldrich) before use. The final water content was < 10 ppm (determined by Karl Fischer titration). Lithium bis(trifluoromethane)sulfonimide (LiTFSI, Aldrich) was dried at 70 ^{o}C under vacuum over several days. Phenol was obtained from Aldrich. The prepared electrolyte solutions contain < 10 ppm water content (determined by Karl Fischer titration). High purity N5.5 O_2 (BOC) was used in all measurements. O_2 gas flow was further dried by an in-line moisture trap filled with activated 3 Å molecular sieves. All materials were stored in an Ar-filled glove box.

Cyclic voltammetry (CV) was performed using a VMP3 electrochemical workstation (Biologic) and a multi-necked, air-tight glass cell within a glove box. The measurements were carried out at room temperature and IR correction was used. 2 mm diameter polycrystalline glassy carbon (GC) disks (BAS Inc.) were employed as the working electrodes. A platinum wire served as the counter electrode and a partially oxidized $LiFePO_4$ composite electrode behind a Vycor frit served as the reference electrode, as described previously.[1]

Swagelok Li-O_2 cells were constructed as described previously.[2] Binder-free gas diffusion layers (GDL, H2315, Quintech) served as the O_2 electrode. GDLs were heated under an Ar:H_2 (95:5, v:v) atmosphere at 900 ^{o}C for three hours. The porosity of the GDLs is ~80 % and the Brunauer-Emmett-Teller surface area is below 1 m^2 g^{-1}.[3] A piece of GDL (4 mm in diameter) served as the cathode, a glass fibre filter (Waterman) as the separator and a partially oxidized $LiFePO_4$ electrode was used as the anode. The two-phase Li_xFePO_4 has a fixed potential of 3.45 V *vs.* Li^+/Li. 150 µl of 30 mM Phenol or Perchloric acid - 1 M LiTFSI in TEGDME served as the electrolyte. All cell components were dried at 90 ^{o}C under vacuum prior to use. Assembled cells were placed in glass tubes, which were filled with dried O_2 inside the glove box. Cells were discharged inside an Ar-filled glove box.

Characterisations of discharged electrodes

For post-cycling characterisation, the cells were dissembled in a glovebox and the cathodes were rinsed with DME and dried prior to further characterisation. The morphology of discharge electrodes were observed by FE-SEM using a Zeiss-Merlin. PXRD was carried out with a Rigaku X-ray diffractometer in an air-sensitive holder. FTIR spectra were measured with a Thermo IR spectrometer (Nicolet 6700) in a N_2-filled glove box. A DEMS cell was constructed as described previously.[4] A GDL served as working electrode and a partially oxidised $LiFePO_4$ composite electrode served as the anode in place of a protected lithium anode. The electrolyte solution was 30 mM Phenol-1 M LiTFSI in TEGDME. A continuous 95% O_2 / 5% Ar gas flow was purged through the cell as a carrier gas at a flow rate of 0.5 ml min^{-1}.

The quantity of Li_2O_2 formed was determined by UV-vis spectrometry (Thermo Evolution 200) using a UV-vis titration method reported previously.[5] The unwashed discharged electrode and separators

were added to a vial containing a known amount of water; Li_2O_2 reacts with water to produce H_2O_2 in solution. 1 ml of this solution was mixed with 2 ml of 2 % $TiOSO_4$ dissolved in 1 M H_2SO_4 solution and a yellowish complex $[Ti(O_2)]^{2+}$ (λ_{max}= 405 nm) was formed. The UV-vis absorption spectrum of the solution was measured and compared to a calibration curve, which was obtained by measuring solutions with known amounts of commercial Li_2O_2 (Aldrich). The purity of commercial Li_2O_2 was determined by titration using $KMnO_4$ and this was taken into account when constructing the calibration curve.

ICPMS

Dry Li_2O_2 powder was added into the solutions containing 0, 10, 25, 50 and 100 mM phenol in TEGDME and stirred for 16 hours. The emulsion was then centrifuged, and liquid phase extracted. The solvent was slowly removed by evaporation under vacuum at a temperature of 70 C°. Finally, 1 M nitric acid solution was added prior to elemental analysis by inductive coupled plasma mass spectroscopy.

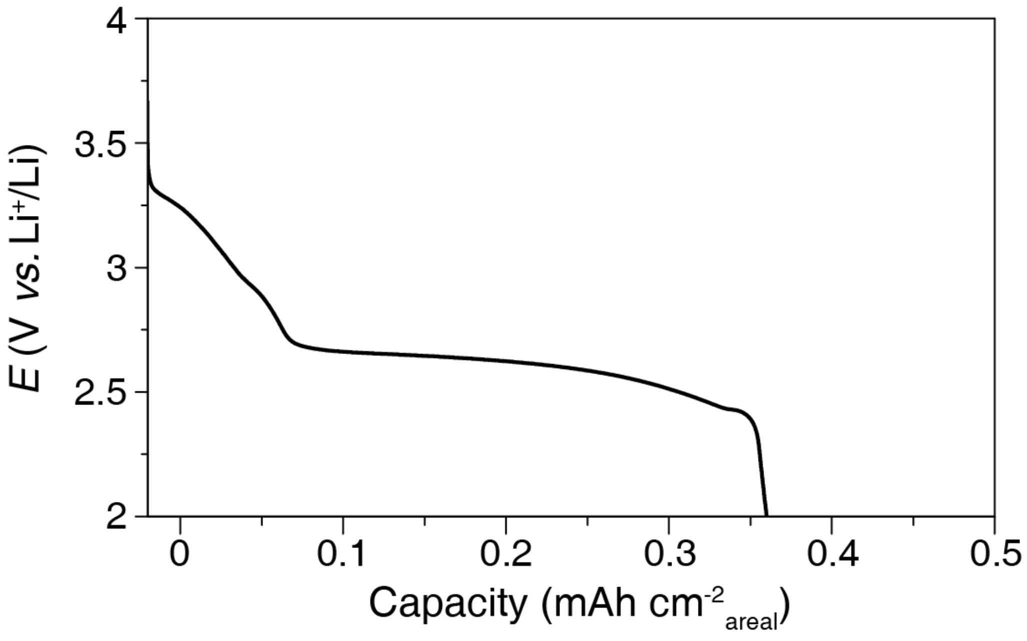

Figure S1. Load curve of O_2 reduction at a gas diffusion electrode discharged in 1 M LiTFSI in TEGDME with 30 mM $HClO_4$.

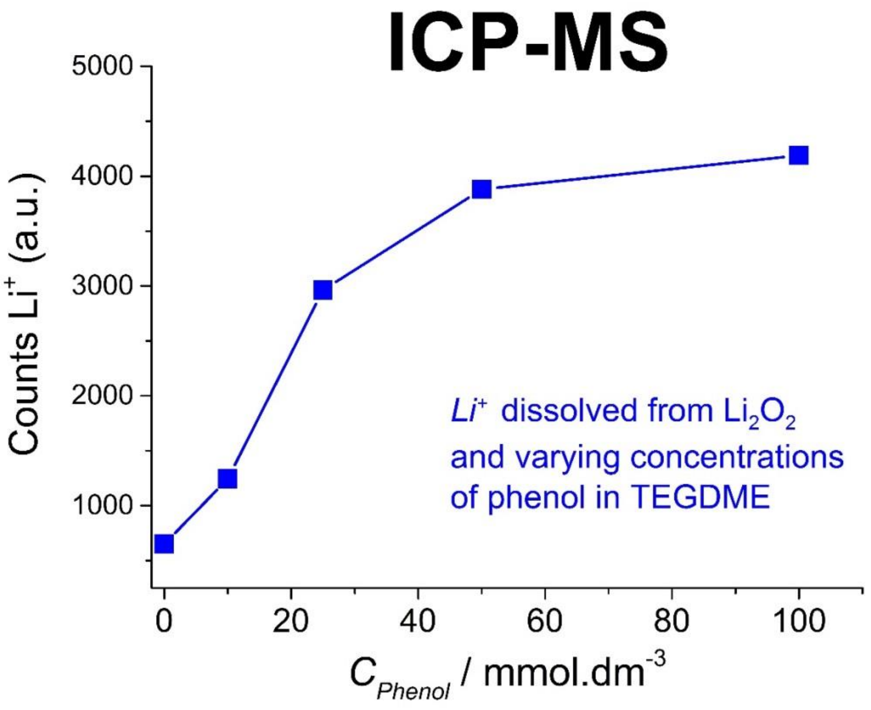

Figure S2. Quantitative analysis of the total amount of Li^+ dissolved in TEGDME solutions containing varying concentrations of phenol as the additive.

References

[1] aL. Johnson, C. Li, Z. Liu, Y. Chen, S. A. Freunberger, P. C. Ashok, B. B. Praveen, K. Dholakia, J.-M. Tarascon, P. G. Bruce, *Nat. Chem.* **2014**, *6*, 1091-1099; bX. Gao, Y. Chen, L. Johnson, P. G. Bruce, *Nat. Mater.* **2016**, *15*, 882–888.

[2] M. M. Ottakam Thotiyl, S. A. Freunberger, Z. Peng, Y. Chen, Z. Liu, P. G. Bruce, *Nat. Mater.* **2013**, *12*, 1050-1056.

[3] P. Hartmann, C. L. Bender, M. Vracar, A. K. Durr, A. Garsuch, J. Janek, P. Adelhelm, *Nat. Mater.* **2013**, *12*, 228-232.

[4] Y. Chen, S. A. Freunberger, Z. Peng, F. Barde, P. G. Bruce, *J. Am. Chem. Soc.* **2012**, *134*, 7952-7957.

[5] aK. U. Schwenke, M. Metzger, T. Restle, M. Piana, H. A. Gasteiger, *J. Electrochem. Soc.* **2015**, *162*, A573-A584; bP. Hartmann, C. L. Bender, J. Sann, A. K. Durr, M. Jansen, J. Janek, P. Adelhelm, *Phys. Chem. Chem. Phys.* **2013**, *15*, 11661-11672.
